# Fulvestrant-3-Boronic Acid (ZB716) Demonstrates Oral Bioavailability and Favorable Pharmacokinetic Profile in Preclinical ADME Studies

**DOI:** 10.3390/ph14080719

**Published:** 2021-07-26

**Authors:** Jiawang Liu, Nirmal Rajasekaran, Ahamed Hossain, Changde Zhang, Shanchun Guo, Borui Kang, Hunsoon Jung, Hongjoong Kim, Guangdi Wang

**Affiliations:** 1 RCMI Cancer Research Center, Xavier University of Louisiana, New Orleans, LA 70125, USA; jliu90@uthsc.edu (J.L.); hahamed@xula.edu (A.H.); czhang1@xula.edu (C.Z.); sguo@xula.edu (S.G.); bkang@xula.edu (B.K.); 2 College of Pharmacy, University of Tennessee Health Sciences Center, Memphis, TN 38163, USA; 3 Enhancedbio Inc., 19 Sangwon-gil, Seongdong-gu, Seoul 04779, Korea; nirmalraj@enhancedbio.com (N.R.); hunsoonjung@enhancedbio.com (H.J.); 4 Zenopharm Inc., 1441 Canal Street, New Orleans, LA 70112, USA

**Keywords:** selective estrogen receptor degrader, ZB716, pharmacokinetics, oral bioavailability, breast cancer, fulvestrant

## Abstract

Fulvestrant-3-boronic acid (ZB716), an oral selective estrogen receptor degrader (SERD) under clinical development, has been investigated in ADME studies to characterize its absorption, metabolism, and pharmacokinetics. ZB716 was found to have high plasma protein binding in human and animal plasma, and low intestinal mucosal permeability. ZB716 had high clearance in hepatocytes of all species tested. ZB716 was metabolized primarily by CYP2D6 and CYP3A. In human liver microsomes, ZB716 demonstrated relatively low inhibition of CYP1A2, 2C8, 2C9, 2C19, 2D6, and 3A4 (when testosterone was used as the substrate), and no inhibition of CYP2B6 and 3A4 (when midazolam was used as the substrate). In assays for enzyme activity, ZB716 induced CYP1A2, 2B6, and 3A4 in a concentration-dependent manner. Single-dose and repeated-dose pharmacokinetic studies in rats and dogs showed oral bioavailability, dose-proportional drug exposure, and drug accumulation as measured by maximum concentration and area under the concentration–time curve (AUC).

## 1. Introduction

Fulvestrant was approved for second-line endocrine treatment in 2002 [1], and recently as a first-line therapy for advanced or metastatic estrogen receptor-positive (ER+), human epidermal growth factor receptor 2-negative (HER2-) breast cancer [2,3]. Fulvestrant acts as a full estrogen receptor (ER) antagonist that degrades the receptor protein, conferring clinical efficacy in treating patients with progressive disease while on tamoxifen or aromatase inhibitors. However, fulvestrant is not orally bioavailable, and its poor pharmacokinetic properties and insufficient drug exposure are believed to limit clinical response, which could be further improved. Efforts to increase fulvestrant exposure through multiple clinical trials between 2005 and 2009 led to subsequent approval of a higher dosage regimen in 2010 [4]. Still, clinical studies indicate that at the maximum injectable limit of 500 mg, the therapeutic effect has not reached its optimal level [5]. This unmet clinical need has driven the continued search for an ER-degrading antiestrogen that could achieve greater drug exposure through high potency and oral bioavailability.

In the past 10 years, over 13 oral SERDs have entered clinical trials to test their safety and efficacy in ER+ breast cancer patients [6]. These oral SERDs are nonsteroidal small molecules characterized by an ER-binding motif and a side chain featuring either an acrylic acid or an amino base group that confer antiestrogenic and ER-degrading activities [7]. In preclinical studies evaluating efficacy and mode of action, the oral SERD candidates are compared with the benchmark fulvestrant—the only clinically approved SERD. In most cases, the oral SERDs are found to be comparable, but not superior, to fulvestrant in ER binding, ER degradation, tissue selectivity, and inhibiting xenograft tumor growth. It is hoped that the oral SERDs, when given to patients, would derive greater efficacy via higher drug exposure than fulvestrant.

**Figure 1:**
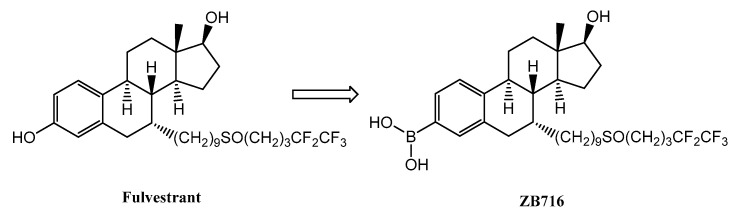
Structure of fulvestrant and ZB716.

The main challenge that oral SERDs seek to overcome, therefore, is the pharmacokinetic limitations of fulvestrant, but not its potency as a pure antiestrogen and ER degrader. If fulvestrant were orally bioavailable and the level of drug exposure could be increased by oral dosage, its clinical efficacy could be conceivably enhanced significantly. In reality, glucuronidation of fulvestrant by UDP-glucuronosyltransferase (UGT) enzymes expressed in both the intestine and liver may effectively inactivate and eliminate the drug before it reaches systemic circulation [8,9,10]. To circumvent the first pass clearance that limits fulvestrant’s oral absorption, we implemented a solution where the 3-OH group of fulvestrant is replaced by a boronic acid group to obtain fulvestrant-3-boronic acid (ZB716) (Figure 1) [11]. The rationale for this chemical modification was based on our research findings [12,13,14,15], where boronic bioisosteres can significantly reduce the first-pass metabolism of phenolic compounds. Our studies confirmed that ZB716 retains full binding affinity of the steroidal moiety of fulvestrant, while minimizing glucuronidation and sulfation [11,12,13]. We found that ZB716 acted as a pure antiestrogen and ER degrader in ER+ breast cancer cells, and potently inhibited tumor growth in clinically relevant xenograft breast cancer models [12].

We report preclinical studies in support of the investigational new drug (IND) application for a phase 1 clinical trial of ZB716. These studies include in vitro ADME investigations, and pharmacokinetic (PK) and toxicokinetic (TK) studies in rodents and dogs. The species and strains used in the present studies reflected those employed in the toxicological testing of ZB716, in order to enable meaningful assessment of the exposure levels in the toxicity studies, and provided confidence in the conclusions drawn regarding the safety of ZB716 in humans. Analysis in support of the toxicokinetic (TK) evaluations in pivotal repeat-dose toxicity studies was performed in compliance with the US FDA GLP Regulations for Nonclinical Laboratory Studies (21 CFR Part 58).

## 2. Results

### 2.1. Absorption

#### 2.1.1. Pharmacokinetics/Toxicokinetics after a Single Dose of ZB716 in Rats

The single-dose PK and relative bioavailability of ZB716 were characterized in male and female Sprague Dawley rats (*n* = 3) after oral administration of 30, 100, and 400 mg/kg/day as suspension (20% PG, 5% Solutol, and 75% of 40% HP-β-CD in water). The bioavailability of ZB716 was also investigated in this study after administration of solution (5% DMSO, 20% PEG400, and 75% of 40% HP-β-CD in saline) using dose levels of 2 mg/kg/day (IV) and 6 mg/kg/day (PO). Plasma samples were collected at various times through to 24 h post-dose, and analyzed for ZB716.

Time to reach maximum plasma concentration (Tmax) was observed mostly 0.5–2 h post-dose following single oral administration of ZB716. Systemic exposure to ZB716, expressed as the maximum plasma concentration (C_max_) and area under the curve up to the last measurable concentration (AUC_last_) values, increased with dose in a close to or greater than dose-proportional manner from 6 to 30 mg/kg/day, and increased with dose in a less than dose-proportional manner from 30 to 100 mg/kg/day, and from 100 to 400 mg/kg/day. By comparing exposure in animals given 6 mg/kg/day orally and animals given 2 mg/kg/day IV, bioavailability was 6.36% in males and 5.98% in females. Group mean TK parameters are shown in Table 1.

#### 2.1.2. Pharmacokinetics/Toxicokinetics after Repeated Doses of ZB716 in Rats

**Figure 2:**
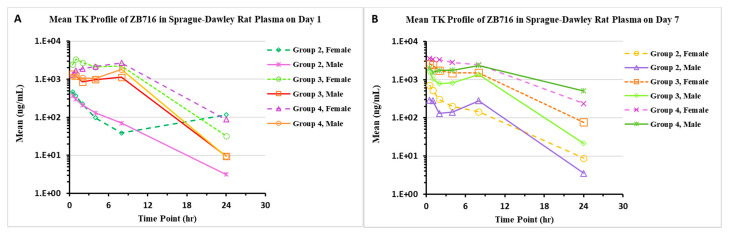
Pharmacokinetic profiles of ZB716 on day 1 (**A**) and day 7 (**B**) in Sprague Dawley rat plasma. Groups 2, 3, and 4 were given ZB716 at 10 mg/kg/day, 100 mg/kg/day, and 400 mg/kg/day, respectively.

Groups of Sprague Dawley rats (*n* = 5/sex/group; *n* = 3/sex/TK group) were orally administered ZB716 (20% PG, 5% Solutol, and 75% of 40% HP-β-CD in DI water) at dose levels of 10, 100, and 400 mg/kg/day for 7 days. At the end of the study, the T_max_ of ZB716 was mostly 0.5–1 h post-dose in animals given 10 and 100 mg/kg/day, and up to 8 h post-dose in animals given 400 mg/kg/day on both day 1 and day 7. Systemic exposure, expressed as C_max_ and AUC_last_ values, increased with dose in a less than dose-proportional manner on days 1 and 7 (Figure 2). A slight gender difference was observed; generally, systemic exposures were higher in females than in males (male/female AUC_last_ ratio = 0.442:0.883). Accumulation was observed only in male animals given 400 mg/kg/day. Group mean TK parameters are shown in Table 2.

The PK of ZB716 was investigated in a 4-week repeat-dose toxicity study in rats. Groups of Sprague Dawley rats (*n* = 10/sex/group; *n* = 3/sex/TK group) were orally administered ZB716 (5% EtOH, 15% PG, 5% Solutol, and 75% of 40% HP-β-CD in DI water) at dose levels of 25, 100, and 400 mg/kg/day for 4 weeks. The plasma concentration of ZB716 was measured at various times through to 24 h post-dose on days 1 and 28. The exposure generally increased with dose in a less than or close to dose-proportional manner on both days 1 and 28. A gender difference was noted with higher exposure at all dose levels for females on day 1, and for females given 25 mg/kg/day on day 28. No accumulation was noted after 28 days of dosing, and a trend for decreased exposure was noted in females given 100 and 400 mg/kg/day after repeat dosing. Systemic exposure data, expressed as C_max_ and AUC_last_ for ZB716, are summarized in Table 3. There was no test-article-related mortality or moribundity during the study.

#### 2.1.3. Pharmacokinetics/Toxicokinetics after a Single Dose of ZB716 in Dogs

The single-dose PK of ZB716 was characterized in beagle dogs after oral administration as a suspension (20% PG, 5% Solutol, and 75% of 40% HP-β-CD in water), using dose levels of 30 and 100 mg/kg/day. The relative bioavailability of ZB716 was also investigated after IV administration of solution (5% DMSO, 20% PEG400, and 75% of 40% HP-β-CD in saline) using a dose level of 2 mg/kg/day, and oral administration of 6 mg/kg/day in solution (5% DMSO, 20% PEG400, and 75% of 40% HP-β-CD in saline). All groups had one male and one female animal. Plasma samples were collected at various timepoints, through to 24 h post-dose, and analyzed for ZB716. Systemic exposure to ZB716, expressed as C_max_ and AUC_last_ values, increased with dose in a greater than dose-proportional manner in the male animal, and in a less than dose-proportional manner in the female animal, from 6 to 30 mg/kg/day.

The systemic exposure increased with dose in a less than dose-proportional manner from 30 to 100 mg/kg/day for both male and female animals. No obvious gender difference was observed. When comparing exposure in animals given 6 mg/kg/day orally and animals given 2 mg/kg/day IV, bioavailability was 6.18% in males and 6.74% in females. The PK parameters derived from these studies are presented in Table 4.

#### 2.1.4. Pharmacokinetics/Toxicokinetics after Repeated Doses of ZB716 in Dogs

The TK of ZB716 was investigated following once-daily administration via oral gavage for 7 consecutive days. Beagle dogs (*n* = 1/sex) were orally administered ZB716 at dose levels of 100 and 200 mg/kg/day. Following 7-day repeat oral administration, the T_max_ of ZB716 was observed mostly at 0.5–2 h post-dose.

**Figure 3:**
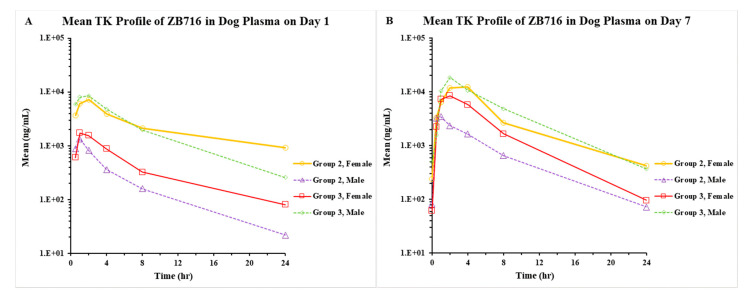
Pharmacokinetic profiles of ZB716 on day 1 (**A**) and day 7 (**B**) in beagle dog plasma. Groups 2 and 3 were given 100 mg/kg/day and 200 mg/kg/day of ZB716, respectively.

The TK of ZB716 was investigated following once-daily administration via oral gavage for 7 consecutive days. The C_max_ and AUC_last_ values of ZB716 increased with dose in a greater than dose-proportional manner. However, the exposure in the female given 100 mg/kg/day was higher than that in the female given 200 mg/kg/day on days 1 and 7 (Figure 3). During the study, females had higher exposure at 100 mg/kg/day, and males had higher exposure at 200 mg/kg/day. Accumulation was observed at all doses after 7 days of dosing. The PK parameters derived from this study are presented in Table 5.

The TK of ZB716 was investigated in a 4-week repeat-dose toxicity study in dogs. Groups of dogs (*n* = 3/sex/group) were orally administered ZB716 (5% EtOH, 15% PG, 5% Solutol, and 75% of 40% HP-β-CD in DI water) at dose levels of 25, 100, and 200 mg/kg/day for 4 weeks. The plasma concentration of ZB716 was measured at various times up through to 24 h post-dose on days 1 and 28. Following oral administration of ZB716 at 25, 100, and 200 mg/kg/day, the exposure generally increased with dose in a less than dose-proportional manner on both days 1 and 28, except that the values did not increase with dose from 100 to 200 mg/kg/day in females on day 1, or in males on day 28. The lower exposure in females given 200 mg/kg/day on day 1 was likely due to emesis noted in all females in this group. Higher exposure was noted in females given 25 mg/kg/day on days 1 and 28, and lower exposure was noted in females given 200 mg/kg/day on day 1. Accumulation was noted at all dose levels after 28 days of dosing, with a day 28/day 1 AUC ratio of 2.06:5.49, except for males given 400 mg/kg/day. The PK parameters derived from this study are presented in Table 6. There was no test-article-related mortality or moribundity during the study.

### 2.2. Distribution

A range of in vitro studies have been performed to investigate the binding of ZB716 to serum and plasma proteins, and to investigate ZB716’s interactions with a range of cellular transporters.

#### 2.2.1. Plasma Protein Binding

The in vitro binding of ZB716 to plasma proteins was determined by equilibrium dialysis technique in mouse, rat, dog, monkey, and human plasma (EDTA-K2 as anticoagulant), over the concentration range of 0.1–10 μM. The free concentration of ZB716 was below the lower limit of quantitation at 0.1, 1, and 10 μM in human, monkey, dog, rat, and mouse plasma. The results showed that ZB716 had a high protein binding in human, monkey, dog, rat, and mouse plasma (Table 7).

#### 2.2.2. Intestinal Mucosal Permeation

The bidirectional permeability and absorption mechanism of ZB716 was evaluated across Caco-2 cell monolayers. As shown in Table 8, the efflux ratio of ZB716 at concentrations of 1, 5, and 15 μM was >0.935, >4.55, and 2.87, respectively. The apparent permeability P_app_ (A to B) was <0.778 cm/s × 10^−6^, <0.159 cm/s × 10^−6^, and 0.188 cm/s × 10^−6^, respectively. The concentration of the receiver was below the lower limit of quantitation at 1 and 5 μM, and the exact value of P_app_ and the efflux ratio could not be calculated. According to the results at 15 μM, ZB716 is a compound with low permeability, and may be a substrate of efflux transporters.

### 2.3. Metabolism

#### 2.3.1. In Vitro Metabolism Including P450 Studies

To determine which CYP enzymes are responsible for the metabolism of ZB716, heterologously expressed recombinant human CYP1A2, 2A6, 2B6, 2C8, 2C9, 2C19, 2D6, 2E1, 3A4, and 3A5 isoforms, along with human liver microsomes, were treated with ZB716 in the presence and absence of CYP-specific chemical inhibitors.

The phenotyping results (Table 9) indicated that CYP2D6 and 3A may be the main CYP isoforms involved in the metabolism of ZB716. The metabolism of ZB716 was observed in incubations with heterologously expressed CYP2D6, 3A4, and 3A5 enzymes, with Cl_int_ values of 0.773, 2.35, and 6.86 μL/min/pmol, respectively. In human liver microsome incubations, with and without specific inhibitors for 2D6 and CYP3A, the remaining percentage after incubation with the CYP2D6 and 3A inhibitors quinidine and ketoconazole was 65.6% and 74.4%, respectively (Table 10). The inhibition ratios (%) were 74.7% and 88.2%. Based on the data on other isoforms, CYP1A2, 2A6, 2B6, 2C8, 2C9, 2C19, and 2E1 may be also involved—to a much lesser extent—in ZB716 metabolism.

#### 2.3.2. Enzyme Inhibition

The potential for ZB716 to inhibit human CYP1A2, CYP2B6, CYP2C8, CYP2C9, CYP2C19, CYP2D6, and CYP3A4 was investigated using human liver microsomes and specific probe substrates (Table 11). Over the test concentration range of 0.05–15 μM, the inhibition of CYP1A2, 2C8, 2C9, 2C19, 2D6, and 3A4 (when testosterone was used as the substrate) activity by ZB716 was observed with the mean IC_50_ values of >15.0, 6.27, 4.77, 2.20, 12.5, and 11.2 μM, respectively (Table 11). The inhibition of CYP2B6 and 3A4 (when midazolam was used as the substrate) by ZB716 was not observed. The inhibitory potential of ZB716 on human CYP enzyme activities appears relatively low. Furthermore, ZB716 showed some inhibitory potential for CYP2C8, 2C9, and 2C19.

#### 2.3.3. Enzyme Induction

The potential for ZB716 to induce the activity of CYP1A2, 2B6, and 3A4 was investigated in plated cultures of cryopreserved human hepatocytes from three donors at 0.15, 1.5, 5, and 15 μM. As shown in Table 12, based on the enzyme activity, the induction of CYP1A2 was observed in two donors (ZSE and VKB) at concentrations of 1.5–15 μM. The induction fold was 2.09–7.62 compared to the vehicle control, and the induction percentage was 20.3–80.0% of the positive control. The induction of CYP2B6 was observed in all three donors at 1.5–15 μM. The induction fold was 2.98–6.40 compared to the vehicle control, and the induction percentage was 37.4–92.1% of the positive control. However, the induction of CYP3A4 was observed in one donor at the concentrations of 5–15 μM. The induction fold ratio was 3.01–2.92 compared to vehicle control, and the induction percentage was 22.4–21.5% of the positive control. The effect was dose-dependent; the decrease in fold induction at 15 μM may be caused by the slight cytotoxicity, and so ZB716 may have an induction effect on CYP1A2, 2B6, and 3A4.

#### 2.3.4. Metabolic Stability

Metabolic stability of ZB716 was assessed at a single concentration of 1 μM) at t = 0 and at t = 120 min. The calculated in vitro Cl_int_ of ZB716 incubated with human, monkey, dog, rat, and mouse hepatocytes was 39.7 μL/min/10^6^ cells, 50.3 μL/min/10^6^ cells, 51.9 μL/min/10^6^ cells, 48.7 μL/min/10^6^ cells, and 40.3 μL/min/10^6^ cells, respectively, with estimated half-lives of 34.9, 27.6, 26.7, 28.4, and 34.4 min, respectively. The results indicate that ZB716 had high clearance in hepatocytes of all species tested (Table 13).

## 3. Discussion

Systemic exposure profiles, based on maximum concentration (C_max_) and area under the concentration–time curve (AUC), were evaluated in rats and dogs. In rats, exposure increased with dose in a close to dose-proportional manner in males, and in a greater than dose-proportional manner in females, at lower doses (6 to 30 mg/kg), and in a less than dose-proportional manner in both sexes at higher doses (30 to 100 mg/kg). No other gender differences were noted in single-dose PK in rats. In dogs, exposure increased in a greater than dose-proportional manner in males in one case, and in a less than dose-proportional manner in both sexes in another study. No other gender differences were noted in single-dose PK in dogs. Repeat-dose TK was evaluated in Sprague Dawley rats and beagle dogs. In rats, exposure generally increased in a less than dose-proportional manner. Some evidence of a gender difference was observed, where females showed higher exposure than males. Accumulation was noted in males at 400 mg/kg/day for 7 days, which was not observed after 28 days of dosing. In dogs, exposure generally increased with dose in a less than dose-proportional manner. Although some gender differences were noted for area under the concentration–time curve from 0 to the time of the last measurable concentration (AUC_last_) ratios on specific days and at specific doses, no clear pattern emerged. Accumulation was evident with repeat dosing. Repeat oral administration of ZB716 once daily in beagle dogs at doses up to 200 mg/kg/day for 28 consecutive days was tolerated. Abnormal clinical signs in animals given 100 mg/kg/day, and lower body weight gains in animals given 200 mg/kg/day, were also observed. Alterations in clinical chemistry parameters and microscopic findings were noted in the liver and thymus. These findings were fully or partially recovered by the end of the 14-day recovery period.

The absorption of ZB716 appears to be relatively rapid, with a C_max_ in general at 0.5–8 h in rats and dogs. At higher doses, the exposure tends to be less than dose-proportional. In both rats and dogs, the plasma levels of ZB716 were generally higher in females than in males.

The in vitro metabolic stability of ZB716 in hepatocytes of five different species all indicated extensive metabolism and rapid clearance, predicting high clearance in vivo. Compared to the positive control verapamil—an oral drug—ZB716 showed a longer half-life and a lower degree of hepatic clearance, consistent with the moderate oral bioavailability of ZB716. As a highly lipophilic compound, ZB716 showed high plasma protein binding in mouse, rat, dog, monkey, and human plasma, with >99% bound at 1 µM. The metabolic pattern obtained in liver microsomes is overall similar in rats, dogs, and humans. The hydrophobic and slightly acidic properties of ZB716 contribute to its high binding to plasma proteins, which likely increases its solubility in plasma. A connection with the plasma proteins protects ZB716 from oxidation, potentially increasing its half-life in vivo.

While CYP2D6 and CYP3A were the primary metabolizing enzymes for ZB716, several CYP isozymes–including CYP1A2, CYP2D6, CYP3A, 2A6, 2B6, 2C8, 2C9, 2C19, and 2E1—were also involved in the metabolism of ZB716, suggesting that drug interactions by single CYP inhibitors are unlikely to modulate the exposure to ZB716 and its metabolites. In human liver microsomes, ZB716 demonstrated relatively low inhibition of CYP1A2, 2C8, 2C9, 2C19, 2D6, and 3A4 (when testosterone was used as the substrate), and no inhibition of CYP2B6 and 3A4 (when midazolam was used as the substrate). Although ZB716 showed the potential to induce CYP1A2, 2B6, and 3A4, the overall results suggest a relatively low risk for drug interactions involving these enzymes in vivo. At therapeutically effective levels of ZB716 (~100 nM), inhibition of CYP enzymes by ZB716 is unlikely.

In summary, the present results support the nonclinical toxicological program of ZB716, as well as the clinical development of ZB716 as an oral SERD for phase 1/2 clinical study in breast cancer patients.

## 4. Materials and Methods

### 4.1. Materials

Amodiaquine, diclofenac sodium, dextromethorphan, furafylline, tranylcypromine, ticlopidine, montelukast, sulfaphenazole, ketoconazole, omeprazole, atenolol, digoxin (European Pharmacopoeia reference standard), and verapamil were purchased from Sigma-Aldrich. Rifampicin and phenobarbital were purchased from Solarbio, China. Phenacetin, bupropion, flumazenil and minoxidil were purchased from the National Institutes for Food and Drug Control (NIDFC), China. Midazolam was procured from Cerilliant, TX, USA. Mephenytoin and N-3-benzylnirvanol were purchased from Toronto Research Chemicals (TRC), ON, Canada. Quinidine was procured from TCI, Japan. Testosterone (#A0256659, Acros) and acetonitrile were received from Fischer Scientific. ZB716 was synthesized as described in our earlier report [11].

### 4.2. Animals

Four-to-six-week-old Sprague Dawley rats (Crl: CD (SD)) (SPF/VAF) were purchased from Beijing Vital River Laboratory Animal Technology, Co. Ltd., Beijing, China). Six-month-old beagle dogs were purchased from Beijing Marshall Biotechnology Co., Ltd. Animals were quarantined/acclimated for 2–6 weeks prior to dose initiation. Food was provided ad libitum, except during overnight fasting prior to blood collection for clinical chemistry and necropsy. Water was provided ad libitum. The feed was analyzed for concentrations of specified heavy metals and nutrient components. The water was routinely analyzed for specific microbes and contaminants, including total dissolved solids, inorganic matter, total chlorinated organic chemicals, and heavy metals. No contaminants were detected in the feed, bedding, or water at levels that might have interfered with the outcome of the study. Records of all dogs vaccinated against distemper, hepatitis, leptospirosis, parvo, parainfluenza, and rabies, as well as prophylactic treatments for parasites, were provided by the vendor. Pharmaron’s Institutional Animal Care and Use Committee (IACUC) reviewed the protocol and approved the animal care and use application. All study activities were in accordance with Pharmaron’s IACUC policies and procedures.

### 4.3. Metabolic Stability

Hepatocytes of humans (#X008001), dogs (#M00205), rats (#M00005), and mice (#M005052) were purchased from BioIVT, NY, USA. Monkey hepatocytes were procured from RILD, Shanghai, China. The metabolic stability of ZB716 in human, monkey, dog, rat and mouse hepatocytes was investigated by incubating ZB716 with human, monkey, dog, rat, and mouse hepatocytes (0.5 × 10^6^ cells/mL), or with incubation medium only. The incubations were carried out at a test concentration of 1 μM at 37 °C, over a total incubation period of 120 min. Verapamil was used as the positive control substrate for metabolism across species. Timepoints were taken at 0, 15, 30, 60, 90, and 120 min by adding acetonitrile with IS to stop the reaction. The percentage of the parent drug remaining, clearance, and t_1/2_ were calculated (the calculation details are available in the Supplementary Materials).

### 4.4. CYP Induction

To investigate the potential of ZB716 to induce the expression and activity of CYP1A2, CYP2B6, and CYP3A4 in plated cultures, cryopreserved human hepatocytes from 3 different donors (QBU, ZSE, and VKB) were purchased from BioIVT, NY, USA. For CYP induction study, all incubations were performed in triplicate; the test concentrations of ZB716 in the incubation medium were 15, 5, 1.5, and 0.15 μM, and 0.1% DMSO was used as a vehicle control. The dosing medium with ZB716 or control articles was renewed every 24 h. On the last day of the incubation, the concentrations of ZB716 in the medium were measured at 0, 1, 2, 4, 6, and 24 h. After 72-h treatment, the plate was incubated with specific substrates of CYP1A2, CYP2B6, and CYP3A4 for 30 min. The UPLC–MS/MS (SHIMADZU, LC-30AD) equipped with waters Xselect^®^HSS T3 2.5 μm (2.1 × 50 mm) was used to detect the marker metabolites in the enzyme activity assay. The fold-induction enzyme activity was determined using the ratio calculation (the calculation details are available in the Supplementary Materials).

### 4.5. CYP Inhibition

The final concentrations of ZB716 in the incubation system were 0, 0.05, 0.15, 0.5, 1.5, 5, and 15 µM (final organic solvent concentration ≤ 1%). The inhibition of the cytochrome P450 family (CYP1A2, 2B6, 2C8, 2C9, 2C19, 2D6, and 3A4) in human liver microsomes (0.2 mg/mL) in the presence of NADPH was measured as the decrease in the rate of marker metabolites’ formation compared to non-inhibited controls (=100% activity). The marker substrates for CYP1A2, 2B6, 2C8, 2C9, 2C19, 2D6, and 3A4 (CYP3A4-M and 3A4-T) were phenacetin, bupropion, amodiaquine, diclofenac, mephenytoin, dextromethorphan, midazolam, and testosterone, respectively. The concentrations of the isoform-specific metabolites derived from marker substrates were measured by UPLC–MS/MS. The IC_50_ values were calculated from the percentages of the rate of the metabolite formation relative to non-inhibited controls vs. the concentrations of ZB716 or positive control inhibitors. All incubations were performed in triplicate. The mean of the enzyme activity (% of non-inhibited control) for each concentration was plotted against the log inhibitor concentration, and fitted to an IC_50_ curve. The percentage of remaining activity was calculated by Equation (1):

Remaining Activity (%) = Concentration_test article_/Concentration_vehicle_ × 100% (1)

### 4.6. CYP Phenotyping (CYP Enzymes Are Responsible for the Metabolism of ZB716)

Bactosomes prepared from E. coli that heterologously expressed individual human CYP isoforms together with human CYP reductase were obtained from CYPEX LTD (Scotland, UK), and stored at −80 °C prior to use. ZB716 was incubated with heterologously expressed recombinant human CYP1A2, 2A6, 2B6, 2C8, 2C9, 2C19, 2D6, 2E1, 3A4, and 3A5 isoforms. Incubations were carried out with 2 µM of ZB716 and 50 pmol/mL of CYP protein. Timepoints were taken at 0, 5, 10, 15, 20, and 30 min. Substrate disappearance was used to calculate intrinsic clearance (Cl_int_) and half-life (t_1/2_). All incubations were performed in triplicate. ZB716 was incubated at 2 µM with human liver microsomes in the presence and absence of CYP-specific chemical inhibitors. The chemical inhibitors for CYP1A2, 2A6, 2B6, 2C8, 2C9, 2C19, 2D6, and 3A4 were furafylline, tranylcypromine, ticlopidine, montelukast, sulfaphenazole, N-3-benzylnirvanol, quinidine, and ketoconazole, respectively. Timepoints were collected at 0, 15, 30, 45, and 60 min, and the remaining percentage of the parent drug at each timepoint was calculated (the calculation details are available in the Supplementary Materials). All incubations were performed in triplicate.

### 4.7. Intestinal Mucosal Permeation of ZB716

The apical-to-basolateral (A–B) and basolateral-to-apical (B–A) transport of ZB716 in HBSS (25 mM HEPES) with 1% BSA was measured across Caco-2 cell monolayers. Incubations were performed at approximately 37 °C for 120 min, with the functionality of the test system being confirmed using 5 µM digoxin as a positive control substrate. Transport of 1, 5, and 15 µM of ZB716 or control compound was determined by quantifying substrate concentration in the incubation medium of the donor (basolateral) compartment at the beginning of the incubation period, and both the donor (basolateral) and receiver (apical) compartments at the end of the incubation period. The data were used to calculate the apparent permeability (Papp) (the calculation details are available in the Supplementary Materials). All incubations were performed in triplicate, and the integrity of the cell monolayers was confirmed using the marker Lucifer yellow.

### 4.8. Protein Binding

The equilibrium dialysis method was used to investigate the in vitro binding of ZB716 to human, monkey, dog, rat, and mouse plasma proteins. The dialysis phosphate buffer solution (100 mM, pH 7.4) and plasma samples containing 0.1, 1, and 10 μM of ZB716 were added to separate chambers of dialysis wells of the HTDialysis device. The dialysis plate was placed in an incubator at 37 °C with 5% CO_2_ at approximately 100 rpm for 6 h. After incubation, the seal was removed, and 50 μL each of post-dialysis samples was pipetted from both the buffer and plasma chambers into fresh 96-well plates. The samples were analyzed via UPLC–MS/MS. The bound fraction, unbound fraction, recovery (%), and remaining (%) were calculated by Equations (2)–(5):

%Unbound = (Conc._buffer chamber_/Conc._plasma chamber_) × 100 (2)

%Bound = 100% − %Unbound (3)

%Recovery = (Conc._buffer chamber_ + Conc._plasma chamber_)/Conc._Total_ sample × 100 (4)

%Remaining = Conc._6h_/Conc._0h_ × 100 (5)

### 4.9. Pharmacokinetics Analysis

Four-to-six-week-old Sprague Dawley rats (Crl: CD (SD)) (SPF/VAF) were purchased from Beijing Vital River Laboratory Animal Technology, Co. Ltd.). Six-month-old beagle dogs were purchased from Beijing Marshall Biotechnology Co. Ltd. Animals were quarantined/acclimated for 2–6 weeks prior to dose initiation. Both Sprague Dawley rats and beagle dogs were given oral administration of ZB716 (20% PG, 5% Solutol, and 75% of 40% HP-β-CD in water) at different dose levels. After treatment with the test articles, whole blood was collected from each study animal by puncture of the tail vein (0.4 mL of whole blood from rats) and/or the peripheral vein (0.6 mL of whole blood from dogs) into tubes containing K_2_EDTA at each timepoint: pre-dose, and 0.5, 1, 2, 4, 8, and 24 h post-dose. The plasma samples were divided into 2 aliquots (approximately equal in volume) and stored frozen at −75 ± 15 °C within 2 h of the collection until analysis at Pharmaron, China.

The stock solution of ZB716 (5.0 mg/mL) and ZB716-d6 (1 mg/mL) in ACN:DI Water = 1:1 was prepared and stored at −20 ± 5 °C. The desired serial concentrations of working reference analyte solutions were achieved by diluting a stock analyte solution with 50% acetonitrile (0.1% formic acid) in water solution. The bioanalytical LC–MS/MS method was validated for quantitative determination of ZB716 in Sprague Dawley rat plasma and beagle dog plasma, with acceptable selectivity, sensitivity, calibration curve, precision, and accuracy. This method is applicable for the determination of ZB716 in Sprague Dawley rat plasma and beagle dog plasma, with a lower limit of quantification of 20.0 ng/mL, and an upper limit of quantification of 10,000 ng/mL.

Five microliters of diluted supernatant was injected into the LC–MS/MS system (Shimadzu LC30AD HPLC system with a SynergiTM 4 μm Fusion-RP 80 Å (50 × 2 mm) column using gradient elution and a Triple Quad 5500 mass spectrometer) for quantitative analysis. The mobile phases used were 0.1% formic acid in deionized water and 0.1% formic acid in acetonitrile. The pharmacokinetic parameters T_1/2_ (the biological half-life), C_max_ (maximal concentration), T_max_ (time at which C_max_ is observed), and AUC (area under the plasma concentration–time curve) were calculated from the plasma concentration versus time data using WinNonlin (Phoenix^TM^, version 8.1, Mountain View, CA, USA). All PK studies were conducted by Pharmaron in Beijing, China.

## Figures and Tables

**Table 1:** Summary of mean ZB716 PK parameters in rat plasma.

Group	Route	Dose Level (mg/kg/day)	Sex	T_max_ (h) ^a^	C_max_ (ng/mL)	AUC_last_ (h·ng/mL)	Bioavailability (%)
1	IV	2	M	0.0833	5367	4906	NA
			F	0.0833	4530	6443	NA
2	PO	6	M	0.5	321	936	6.36
			F	0.5	347	1155	5.98
3	PO	30	M	2	1723	5410	NA
			F	1	3523	15,691	NA
4	PO	100	M	0.5	3010	12,493	NA
			F	0.5	3590	30,530	NA
5	PO	400	M	0.5	2863	32,608	NA
			F	1	4310	55,568	NA

AUC_last_ = area under the curve up to the last measurable concentration; C_max_ = maximum plasma concentration; F = female; IV = intravenous; PO = per os (by mouth); M = male; NA = not applicable; T_max_ = time to reach maximum plasma concentration. ^a^ T_max_ is median.

**Table 2:** ZB716 7-day repeated-dose PK parameters in rat plasma.

Study Day	Group	Dose Level (mg/kg/day)	Sex	T_max_ (h) ^a^	C_max_ (ng/mL)	AUC_last_ (h·ng/mL)
1	2	10	M	0.5	365	1324
			F	0.5	459	1759
	3	100	M	0.5	1420	11,788
			F	1	3350	26,684
	4	400	M	8	1920	15,150
			F	8	2767	27,885
7	2	10	M	1	322	2255
			F	0.5	710	2822
	3	100	M	0.5	1610	12,884
			F	0.5	2753	20,659
	4	400	M	8	2337	32,740
			F	0.5	3710	37,064

AUC_last_ = area under the curve up to the last measurable concentration; C_max_ = maximum plasma concentration. ^a^ T_max_ is median.

**Table 3:** ZB716 28-day repeated-dose TK parameters in rat plasma.

Study Day	Group No.	Dose Level (mg/kg/day)	Sex	T_max_ (h)	C_max_ (ng/mL)	AUC_last_ (h·ng/mL)
1	2	25	Male	0.5	1039	5405
			Female	1.0	1310	7695
	3	100	Male	1.0	1867	15,708
			Female	1.0	3200	42,161
	4	400	Male	1.0	3397	52,371
			Female	2.0	11,927	120,801
28	2	25	Male	0.5	1313	4748
			Female	1.0	1767	8042
	3	100	Male	0.5	2840	20,087
			Female	1.0	3847	24,525
	4	400	Male	2.0	4063	50,678
			Female	2.0	4853	51,763

AUC_last_ = area under the curve up to the last measurable concentration; C_max_ = maximum plasma concentration; TK = toxicokinetic(s).

**Table 4:** Summary of ZB716 single-dose PK parameters in dog plasma**.**

Group	Route	Dose Level (mg/kg/day)	Sex	T_max_ (h)	C_max_ (ng/mL)	AUC_last_ (h·ng/mL)	Bioavailability (%)
1	IV	2	M	0.0833	2160	3034	NA
			F	0.0833	2710	4312	NA
2	PO	6	M	0.5	157	563	6.18
			F	0.5	213	872	6.74
3	PO	30	M	4	988	5522	NA
			F	1	964	3456	NA
4	PO	100	M	2	1140	7500	NA
			F	0.5	957	5841	NA

AUC_last_ = area under the curve up to the last measurable concentration; C_max_ = maximum plasma concentration; F = female; IV = intravenous; M = male; NA = not applicable; T_max_ = time to reach maximum plasma concentration.

**Table 5:** ZB716 repeated-dose PK parameters in beagle dogs.

Day	Group No.	Dose Level (mg/kg/day)	Sex	T_max_ (h)	Animal No.	C_max_ (ng/mL)	AUC_last_ (h·ng/mL)
1	2	100	M	1	8,135,390	1350	5068
			F	2	8,067,921	7210	55,649
	3	200	M	2	8,121,984	8430	52,222
			F	1	8,135,209	1740	9785
7	2	100	M	1	8,135,390	3430	17,754
			F	4	8,067,921	12,300	81,061
	3	200	M	2	8,121,984	18,300	103,223
			F	2	8,135,209	8420	46,746

AUC_last_ = area under the curve up to the last measurable concentration; C_max_ = maximum plasma concentration. F = female; M = male.

**Table 6:** ZB716 28-day repeated-dose TK parameters in beagle dogs.

Study Day	Group No.	Dose Level (mg/kg/day)	Sex	T_max_ (h)	C_max_ (ng/mL)	AUC_last_ (h·ng/mL)
1	2	25	M	0.5	691	1980
			F	1.0	772	3882
	3	100	M	1.0	1401	4572
			F	1.0	997	4492
	4	200	M	2.0	1037	7876
			F	2.0	583	2718
28	2	25	M	1.0	1386	4327
			F	1.0	1591	8001
	3	100	M	2.0	3337	12,449
			F	2.0	1873	9647
	4	200	M	2.0	2133	11,041
			F	2.0	1807	14,915

AUC_last_ = area under the curve up to the last measurable concentration; C_max_ = maximum plasma concentration; TK = toxicokinetic(s).

**Table 7:** The bound (%), unbound (%), recovery (%), and remaining (%) of ZB716 at 0.1, 1, and 10 μM in human, monkey, dog, rat, and mouse plasma.

Species	Conc. (μM)	Bound (%)	Unbound (%)	Recovery (%)	Remaining (%)
Human	0.1	>95.7	<4.33	<69.1	57.0 ± 7.7
	1	>99.6	<0.392	<64.0	57.9 ± 4.9
	10	>99.96	<0.0108	<74.7	74.5 ± 3.4
Monkey	0.1	>96.4	<3.63	<68.2	65.3 ± 2.8
	1	>99.6	<0.326	<70.0	70.9 ± 4.8
	10	>99.97	<0.0308	<87.2	93.9 ± 10.0
Dog	0.1	>95.6	<4.04	<63.5	39.1 ± 1.8
	1	>99.6	<0.373	<64.9	53.2 ± 4.4
	10	>99.96	<0.0412	<68.7	65.2 ± 4.3
Rat	0.1	>97.7	<2.35	<94.4	83.9 ± 8.2
	1	>99.8	<0.216	<94.9	91.1 ± 2.1
	10	>99.97	<0.0281	<82.1	84.7 ± 2.0
Mouse	0.1	>97.4	<2.63	<96.0	98.5 ± 14.5
	1	>99.7	<0.266	<88.8	92.2 ± 5.7
	10	>99.97	<0.0272	<95.0	97.4 ± 10.7

**Table 8:** Permeability results of ZB716 in Caco-2 cells (mean ± SD, *n* = 3).

Test Article	P_app_ (cm/s × 10^−6^)		Recovery (%)		Efflux Ratio
	A to B	B to A	A to B	B to A	
ZB716 (1 μM) *	<0.778	0.747 ± 0.0316	<66.6	66.5 ± 0.7	>0.935
ZB716 (5 μM)	<0.159	0.820 ± 0.127	<67.0	73.7 ± 1.5	>4.55
ZB716 (15 μM)	0.188 ± 0.021	0.534 ± 0.028	75.7 ± 2.0	84.2 ± 4.8	2.87 ± 0.49
Digoxin (5 μM)	0.687 ± 0.088	14.5 ± 1.68	92.4 ± 1.0	86.0 ± 6.0	21.2 ± 2.0
Atenolol (5 μM)	0.506 ± 0.034	0.635 ± 0.042	89.1 ± 1.2	97.5 ± 0.2	1.26 ± 0.08
Minoxidil (5 μM)	6.47 ± 0.22	7.44 ± 0.47	91.0 ± 1.4	94.4 ± 48	1.15 ± 0.09

* The concentration of the receiver was BLLOQ; the LLOQ was 2 nM.

**Table 9:** The intrinsic clearance (Clint) and half-life (t1/2) of ZB716 incubated with individual recombinant human CYP isoforms (mean ± SD, *n* = 3).

CYP Isoform	Cl_int_ (µL/min/pmol CYP)	t_1/2_ (min)
CYP1A2	0.866 ± 0.120	16.2 ± 2.2
CYP2A6	0.836 ± 0.028	16.6 ± 0.6
CYP2B6	0.343 ± 0.052	41.0 ± 6.8
CYP2C8	1.25 ± 0.14	11.1 ± 1.2
CYP2C9	0.746 ± 0.015	18.6 ± 0.4
CYP2C19	0.646 ± 0.063	21.6 ± 2.0
CYP2D6	0.773 ± 0.142	18.3 ± 3.3
CYP2E1	0.716 ± 0.044	19.4 ± 1.2
CYP3A4	2.35 ± 0.15	5.91 ± 0.39
CYP3A5	6.86 ± 1.12	2.06 ± 0.33

**Table 10:** The remaining percentage of ZB716 at 60 min in human liver microsomes, with and without CYP isoform chemical inhibitors (*n* = 3).

CYP Isoform (Inhibitors)	Remaining Percentage (%) at 15 min	Remaining Percentage (%) at 60 min	Inhibition %
Without Inhibitor	18.7 ± 1.2	5.74 ± 0.52	0.00
CYP1A2 (Furafylline)	8.24 ± 0.80	1.43 ± 0.51	−48.9
CYP2A6 (Tranylcypromine)	14.8 ± 2.5	5.24 ± 2.31	−14.4
CYP2B6 (Ticlopidine)	15.9 ± 0.8	2.09 ± 0.23	−9.46
CYP2C8 (Montelukast)	14.6 ± 2.4	3.41 ± 0.30	−15.1
CYP2C9 (Sulfaphenazole)	10.9 ± 0.1	2.31 ± 0.18	−31.8
CYP2C19 (N-3-benzylnirvanol)	12.8 ± 1.2	3.10 ± 0.44	−22.5
CYP2D6 (Quinidine)	65.6 ± 6.0	3.23 ± 0.63	74.7
CYP3A (Ketoconazole)	74.4 ± 5.8	43.4 ± 2.8	88.2

**Table 11:** The IC_50_ values of ZB716 against CYP1A2, CYP2B6, CYP2C8, CYP2C9, CYP2C19, CYP2D6 and CYP3A4 (*n* = 3).

CYP Isoform	Marker Substrate	IC_50_ (μM) of ZB716
CYP1A2	Phenacetin	>15.0
CYP2B6	Bupropion	No Inhibition
CYP2C8	Amodiaquine	6.27
CYP2C9	Diclofenac	4.77
CYP2C19	Mephenytoin	2.20
CYP2D6	Dextromethorphan	12.5
CYP3A4-M	Midazolam	No Inhibition
CYP3A4-T	Testosterone	11.2

CYP3A4-M—Midazolam used as the substrate of CYP3A4; CYP3A4-T—Testosterone used as the substrate of CYP3A4.

**Table 12:** Induction potential of ZB716 and positive control based on enzyme activity (mean ± SD, *n* = 3).

Donor	CYP	Compound	Conc. (µM)	Fold Induction (Mean ± SD)	Percent of Control (%)
QBU	CYP1A2	Flumazenil	30	1.06 ± 0.11	0.450
		Omeprazole	50	15.0 ± 1.8	-
		ZB716	0.15	1.11 ± 0.05	0.777
			1.5	2.17 ± 0.03	8.38
			5	3.44 ± 0.18	17.4
			15	2.77 ± 0.34	12.6
	CYP 2B6	Flumazenil	30	0.789 ± 0.070	−3.61
		Phenobarbital	1000	6.86 ± 0.36	-
		ZB716	0.15	1.19 ± 0.10	3.16
			1.5	2.16 ± 0.07	19.8
			5	5.21 ± 0.24	71.7
			15	6.40 ± 0.16	92.1
	CYP 3A4	Flumazenil	30	1.39 ± 0.04	4.37
		Rifampicin	10	9.95 ± 0.63	-
		ZB716	0.15	0.797 ± 0.100	−2.26
			1.5	1.43 ± 0.14	4.83
			5	3.01 ± 0.33	22.4
			15	2.92 ± 0.25	21.5
ZSE	CYP 1A2	Flumazenil	30	0.807 ± 0.177	−3.60
		Omeprazole	50	6.37 ± 0.14	-
		ZB716	0.15	1.20 ± 0.22	3.78
			1.5	3.42 ± 0.20	45.0
			5	3.24 ± 0.53	41.6
			15	2.09 ± 0.49	20.3
	CYP 2B6	Flumazenil	30	1.28 ± 0.20	5.23
		Phenobarbital	1000	6.29 ± 0.90	-
		ZB716	0.15	1.14 ± 0.09	2.61
			1.5	3.07 ± 0.36	39.2
			5	2.98 ± 0.74	37.4
			15	1.22 ± 0.42	4.18
	CYP 3A4	Flumazenil	30	1.23 ± 0.09	3.11
		Rifampicin	10	8.36 ± 0.24	-
		ZB716	0.15	0.610 ± 0.043	−5.29
			1.5	0.828 ± 0.082	−2.34
			5	0.513 ± 0.081	−6.62
			15	0.318 ± 0.005	−9.26
VKB	CYP 1A2	Flumazenil	30	0.818 ± 0.032	−2.20
		Omeprazole	50	9.28 ± 0.223	-
		ZB716	0.15	1.24 ± 0.10	2.92
			1.5	2.52 ± 0.17	18.4
			5	7.09 ± 0.78	73.6
			15	7.62 ± 1.63	80.0
	CYP2B6	Flumazenil	30	1.17 ± 0.04	3.77
		Phenobarbital	1000	5.44 ± 0.13	-
		ZB716	0.15	1.10 ± 0.05	2.22
			1.5	1.77 ± 0.13	17.2
			5	4.14 ± 0.06	70.6
			15	4.24 ± 0.47	73.0
	CYP3A4	Flumazenil	30	1.06 ± 0.09	1.16
		Rifampicin	10	5.97 ± 0.52	-
		ZB716	0.15	0.774 ± 0.078	−4.55
			1.5	0.649 ± 0.085	−7.08
			5	1.24 ± 0.18	4.85
			15	1.49 ± 0.21	9.95

**Table 13:** Metabolism information of ZB716 in human, monkey, dog, rat, and mouse hepatocytes (mean, *n* = 3).

Species	Compound	Remaining at 120 min (%)	Estimated In Vitro t_1/2_ (min)	In Vitro Cl_int_ (μL/min/10^6^ cells)	Scaled-Up Cl_int_ (mL/min/kg)
Human	Verapamil	1.57 ± 0.18	20.8 ± 0.6	66.7 ± 2.1	170 ± 5
	ZB716	7.98 ± 0.7	34.9 ± 1.0	39.7 ± 1.1	101 ± 3
Monkey	Verapamil	0.264 ± 0.103	11.35 ± 0.17	122 ± 2	440 ± 6
	ZB716	4.41 ± 0.46	27.6 ± 2.0	50.3 ± 3.6	181 ± 13
Dog	Verapamil	6.02 ± 0.17	30.6 ± 0.4	45.3 ± 0.5	312 ± 4
	ZB716	6.99 ± 0.46	26.7 ± 0.4	51.9 ± 0.7	357 ± 5
Rat	Verapamil	1.24 ± 0.38	18.2 ± 1.8	76.6 ± 8.1	358 ± 38
	ZB716	5.19 ± 0.32	28.4 ± 0.9	48.7 ± 1.5	228 ± 7
Mouse	Verapamil	2.02 ± 0.54	22.0 ± 1.6	63.3 ± 4.8	747 ± 57
	ZB716	6.24 ± 0.58	34.4 ± 0.6	40.3 ± 0.7	476 ± 8
Medium	Verapamil	102	∞	NA	NA
	ZB716	100	∞	NA	NA

## Data Availability

The data presented in this study are available in Pharmaceuticals 2021, 14, 719. https://doi.org/10.3390/ph14080719.

## Supplementary Materials

The following are available online at https://www.mdpi.com/article/10.3390/ph14080719/s1.
