# Supplementary material for: Fulvestrant-3-Boronic Acid (ZB716) Demonstrates Oral Bioavailability and Favorable Pharmacokinetic Profile in Preclinical ADME Studies
Source: Pharmaceuticals (Basel). 2021 Jul 26;14(8):719. doi: 10.3390/ph14080719 (PMC8400955; doi:10.3390/ph14080719)
Supplement: Supplementary file 1 [file pharmaceuticals-14-00719-s001.zip › pharmaceuticals-1296086_supple_v4.pdf]

Supplementary material

Article

Fulvestrant-3-Boronic Acid (ZB716) Demonstrates Oral Bioavailability and Favorable Pharmacokinetic Profile in Pre-clinical ADME Studies

Jiawang Liu[1,2,†], Nirmal Rajasekaran[3,†] ,Ahamed Hossain[1], Changde Zhang[1], Shanchun Guo[1], Borui Kang[1], Hunsoon Jung[3], Hong Joong Kim[3,*], Guangdi Wang [1,4,*]

[1] RCMI Cancer Research Center, Xavier University of Louisiana, New Orleans, LA 70125 ;
J L : jliu90@uthsc.edu; A H: hahamed@xula.edu; C Z: czhang1@xula.edu; S G: @sguo@xula.edu; B K : bkang@xula.edu;
[2] College of Pharmacy, University of Tennessee Health Sciences Center, Memphis, TN
[3] Enhancedbio Inc, 19 Sangwon-gil, Seongdong-gu, Seoul 04779, Republic of Korea
N R: nirmalraj@enhancedbio.com ; H J: hunsoonjung@enhancedbio.com; H J K: joon.kim@enhancedbio.com
[4] Zenopharm, Inc, 1441 Canal Street, New Orleans, LA70112; G W: guangdi.wang@zenopharm.com

† These authors contributed equally to this work.
* Correspondence: G W; guangdi.wang@zenopharm.com (Zenopharm, 1441 Canal Street, New Orleans, LA 70112);
H J K; joon.kim@enhancedbio.com; (Enhancedbio, 19 Sangwon-gil, Seongdong-gu, Seoul 04779, Republic of Korea)

Materials and methods

1.1 Metabolic Stability

The in vitro half-life ($t_{1/2}$) of parent compound was determined by regression analysis of the percent parent disappearance vs. incubation time curve.

The in vitro half-life (in vitro $t_{1/2}$) was determined from the slope value:

$$\textbf{\textit{in vitro}}\ \mathbf{t_{1/2} = 0.693 / k}$$

where k is determined using the equation Percent Parent Remaining = 100%•exp(-k•t)

The in vitro intrinsic clearance (in vitro Clint, in μL/min/106cells) was calculated using the following equation:

$$\textbf{\textit{in vitro}}\ \mathbf{Cl_{int} = kV/N}$$

V = Volume of incubation (0.2 mL); N = Number of hepatocytes per well (0.1 x 10^6cells).

The Scaled-up intrinsic clearance (Scaled-up Cl_{int}, in mL/min/kg) was calculated using the following equation:

$$\mathbf{Scaled\text{-}up\ Cl_{int} = kV/N \times Scaling\ Factor}$$

Scaling factors for in vivo intrinsic clearance prediction are listed below:

**Species**	**Liver Weight (g liver/kg body weight)**	**Hepatocyte Concentration (10^6 cells/g liver)**	**Scaling Factor**	**Liver blood flow (Q, mL/min/kg)**
Human	25.7	99	2544.3	20.7
Monkey	30	120	3600.0	43.6
Dog	32	215	6880.0	30.9
Rat	40	117	4680.0	55.2
Mouse	87.5	135	11812.5	90

1.2 CYP Induction

Enzyme activity

CYP activity was expressed as pmol/min/million cells where pmol was defined as the amount of metabolite formed during the reaction. The number of hepatocytes per well (N) was related to the seeding density. The seeding density was 0.55 million cells/mL, and the number of hepatocytes per well was 0.055 million. The fold-induction enzyme activity was determined by the ratio:

$$\mathbf{Fold\ of\ induction = CYP\ activity_{(induced)} / CYP\ activity_{(vehicle)}}$$

$$\mathbf{\%\ of\ positive\ control} = \frac{(CYP\ activity(induced) - CYP\ activity(DMSO))}{(CYP\ activity(positive\ control) - CYP\ activity(DMSO))} \times \mathbf{100}$$

Based on the enzyme activity, a minimum of 2-fold increase of a response compared to vehicle control and ≥ 20% of the positive control was considered as the cutoff for a positive signal in in vitro induction assay.

1.3 CYP Phenotyping (CYP enzymes are responsible for the metabolism of ZB716)

1.3.1 Intrinsic Clearance (Heterologously Expressed Human Cytochrome P450)

All calculations were carried out using Microsoft Excel. The slope, k, was determined by linear regression of the natural logarithm of percent parent disappearance vs. incubation time curve. Peak areas for test article and internal standard were determined from extracted ion chromatograms. The peak area ratio was used to calculated for the results.

The half-life value ($t_{1/2}\ r_{CYPi}$) was determined from the slope value:

$$\mathbf{t_{1/2}\ r_{CYPi} = 0.693/\ k}$$

Conversion of the $t_{1/2}\ r_{CYPi}$ (in min) into the intrinsic clearance (Clint r_{CYPi} in μL/min/pmol) was done using the following equation:

$$\mathbf{Cl_{int\ rCYPi} = (0.693\ /\ t_{1/2}) \times (\text{Volume of incubation } (\mu L)\ /\ \text{Amount of CYP (pmol)})}$$

1.3.2 For inhibition ratio (%) (Liver microsomes with and without inhibitors)

The slope value, k, was determined by linear regression of the natural logarithm of the remaining percentage of the parent drug vs. incubation time curve. If the turnover was not significant (T-test with $p<0.05$ was not obtained) and the percentage remaining at last time point was ≥ 80%, the k value was not reported.

$$\textbf{Inhibition ratio (\%)} = \frac{((\text{Kwithout inhibitor} - \text{Kwith inhibitor})}{\text{Kwithout inhibitor}} \times \mathbf{100}$$

1.4 Intestinal Mucosal Permeation of ZB716

Apparent permeability (P_{app}, cm/s x 10^{-6}) was calculated for drug transport assays using the following equation:

$$\boldsymbol{P}_{app} = \frac{VA}{Area \times time} \times \frac{[drug]receiver}{[drug]initial,donor}$$

Where: Where: Papp is apparent permeability (cm/s x 10^{-6}); VA is the volume of receiver sides (Ap to Bl is 0.3 mL, Bl to Ap is 0.1 mL); Area is the surface area of the membrane (cm^2); Time is the incubation time (s).

Efflux ratio can be determined using the following equation:

$$\textbf{Efflux Ratio} == \frac{Papp\ (B-A)}{Papp\ (A-B)}$$

Where: Papp (B-A) is apparent permeability from basolateral compartment to apical compartment; Papp (A-B) is apparent permeability from apical compartment to basolateral compartment

Mass balance (recovery %) was determined using the following equation:

$$\textbf{Recovery\%} = = \frac{[drug]\ receiver \times VA + [drug]donor \times VD}{[drug]initial,donor \times VD} \times \mathbf{100}$$

Where: VA is the volume (in mL) in the receiver well (0.1 mL); V_D is the volume (in mL) in the donor well (0.3 mL).

If the Papp (A to B) is less than the Papp (A to B) of atenolol will be considered as low permeability, and if the Papp (A to B) is greater than the Papp (A to B) of minoxidil will be considered as high permeability. If the Papp (A to B) is greater than the Papp (A to B) of atenolol and less than the Papp (A to B) of minoxidil will be considered as moderately permeability. If the efflux ratio of test article is greater than or equal to 2, the test article will be considered as substrate of efflux transporter.
